# Effects of Sub-Lethal Doses of Selenium Nanoparticles on the Health Status of Rats

**DOI:** 10.3390/toxics9020028

**Published:** 2021-02-03

**Authors:** Lenka Urbankova, Sylvie Skalickova, Magdalena Pribilova, Andrea Ridoskova, Pavlina Pelcova, Jiri Skladanka, Pavel Horky

**Affiliations:** 1 Department of Animal Nutrition and Forage Production, Mendel University in Brno, Zemedelska 1, CZ-613 00 Brno, Czech Republic; lenka.urbankova@mendelu.cz (L.U.); magdalena.pribilova@mendelu.cz (M.P.); jiri.skladanka@mendelu.cz (J.S.); pavel.horky@mendelu.cz (P.H.); 2 Department of Chemistry and Biochemistry, Mendel University in Brno, Zemedelska 1, CZ-613 00 Brno, Czech Republic; andrea.ridoskova@mendelu.cz (A.R.); pavlina.pelcova@mendelu.cz (P.P.); 3 CEITEC-Central European Institute of Technology, Mendel University in Brno, Zemedelska 1, CZ-613 00 Brno, Czech Republic

**Keywords:** nanotoxicity, liver enzymes, histopathology, diet, growth performance, glutathione peroxidase, superoxide dismutase, glucose

## Abstract

Selenium nanoparticles (SeNPs) are fast becoming a key instrument in several applications such as medicine or nutrition. Questions have been raised about the safety of their use. Male rats were fed for 28 days on a monodiet containing 0.5, 1.5, 3.0 and 5.0 mg Se/kg. Se content in blood and liver, liver panel tests, blood glucose, total antioxidant capacity (TAC), the activity of superoxide dismutase (SOD) and glutathione peroxidase (GPx) were analysed. Liver and duodenum were subjected to histopathology examination. The weight gain of rats showed no differences between tested groups. Se content in blood was higher in all treated groups compared to the control group. The liver concentration of Se in the treated groups varied in the range from 222 to 238 ng/g. No differences were observed in the activity of AST (aspartate aminotransferase), ALP (alkaline phosphatase) and TAS (total antioxidant status). A significant decrease in ALT activity compared to the control group was observed in the treated groups. GPx activity varied from 80 to 88 U/mL through tested groups. SOD activity in liver was decreased in the SeNP-treated group with 5 mg Se/kg (929 ± 103 U/mL). Histopathological examination showed damage to the liver parenchyma and intestinal epithelium in a dose-dependent manner. This study suggests that short-term SeNP supplementation can be safe and beneficial in Se deficiency or specific treatment.

## 1. Introduction

Selenium is one of the essential microelements that is important for several biochemical reactions in living organisms. Its role in an organism depends on chemical structure. Se can occur in various oxidation states: selenide (-II), selenite (IV), and selenate (VI). Besides the inorganic alterations, Se is bounded in amino acids or their derivates (mostly in the form of selenide). Up to 90% of Se is deposited in selenoproteins in muscle tissue, and the thyroid gland [1]. The most important role of selenium is the antioxidant system’s function, prevention in lipid oxidation and inhibition of DNA damage. Se also contributes to the normal function of the thyroid gland via the iodothyronine deiodinase enzyme converting thyroxin to its active form, triiodothyronine [2].

The therapeutic window for the beneficial effect of Se is very narrow. Toxic potential of Se depends on its chemical form, rate of Se methylation and excretion. Oral median lethal dose (LD_50_) of sodium selenite has been estimated to 7 mg Se/kg body wt., 138 mg Se/kg for selenium sulphides and 6700 mg Se/kg for elemental selenium in rats [3]. Lower selenium concentrations produced chronic symptoms, including a decreased growth rate, a restriction of food consumption, and slight-to-severe pathological lesions. Proposed molecular mechanisms involved in selenium toxicity include interaction with tissue thiols such as glutathione. Selenium-mediated thiol oxidation can produce reactive oxygen species (ROS) and lead to oxidative damage of the organism. Elemental selenium or selenoenzymes do not react with thiols, and, generally, they are non-toxic [4,5].

Progress in nanotechnology has facilitated the production of SeNPs in various forms and sizes. Thanks to the high variability and the ability to adapt nanoparticles, their research is growing rapidly, especially in materials science, chemistry, nanotechnology, physics, and nanomedicine. Development of large-scale production opened the potential of commercial use. There is a large volume of published studies describing selenium nanoparticles’ role in nutrition and dietetics, pharmacy, diagnostic and therapy, agriculture, or environmental studies. The mentioned research is based on the properties of selenium, such as catalytic activity, role in biochemistry and metabolism, antagonistic effect against Ag, As, Cd, Hg, F or Tl, free radicals scavenging, antitumor activity and antimicrobial action [1,6,7]. In recent years, the trend of utilisation of Se for nutritional supplementation is developing with respect to selenium nanoformulation, as we described in our review [8]. It has been observed that the nanoform can penetrate tissues better, can be delivered to targeted tissues or can provide a gradual release [9]. Thus, the nanotoxicity of Se is still under careful consideration. The toxicologic impact of SeNPs has been studied for several kinds of SeNPs in tissue cells [10], rats, mice [11,12], aquaculture [13] or chickens [14]. The main limitation of performed studies is variability in the used type of SeNPs and non-uniform dosing. Questionable is also nano-Se deposition in tissues as well as reactive oxygen species initiation. New pharmacokinetics studies confirmed that the formation of protein corona surrounding nanoparticles leads to a change in nanoparticles’ function [15].

This study aimed to evaluate the effect of sub-lethal doses of selenium nanoparticles (SeNPs) on the health status of Wistar albino rats. Rats have been an extensively used model organism due to genetic and physiological similarities to mammals such as livestock and/or humans. The doses were set up to cover the narrow window from the beneficial to the toxic effect of Se.

## 2. Materials and Methods

### 2.1. Chemicals 

All chemicals, unless noted otherwise, were purchased from Sigma Aldrich (St. Louis, USA). The pH value was measured using inoLab Level 3 (Wissenschaftlich-Technische Werkstatten GmbH; Weilheim, Germany). Deionised water underwent demineralisation by reverse osmosis using the instruments Aqua Osmotic 02 (Aqua Osmotic, Tisnov, Czech Republic). 

### 2.2. Selenium Nanoparticles (SeNPs) 

**Figure 1:**
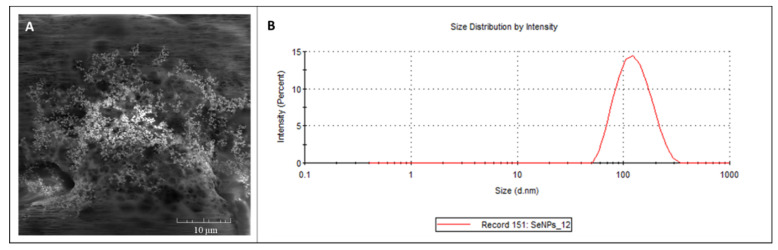
(**A**) SEM documentation and (**B**) size distribution of SeNPs.

Elemental SeNPs (99.99% purity, size: <100 nm) were purchased from Nanografi (Ankara, Turkey. For documentation of the SeNPs’ structure, a scanning electron microscope MIRA (Tescan, Brno, Czech Republic) was used (Figure 1A), equipped with a high brightness Schottky field emitter for low noise imaging fast scanning rates. The SEM is fitted with an In-Beam SE detector. An accelerating voltage of 15 kV and beam currents of about 1 nA give satisfactory results regarding maximum throughput. Magnification 5.00 KX was used.

The mean particle diameter and size distribution (Figure 1B) were determined by dynamic light scattering on a Malvern Zetasizer (NANO-ZS, Malvern Instruments Ltd., Worcestershire, UK).

### 2.3. Animals

The feeding experiments were carried with the approval of the Ethics Commission at the Faculty of AgriSciences, Mendel University, in Brno (Czech Republic, European Union) in accordance with Act No. 246/1992 Coll. on the Protection of Animals Against Cruelty (the experimental project was approved on 20.8.2019; MSMT-15228/219-5). The laboratory rats of Wistar albino strain (males) were used as model animals. The average initial weight of rats was 156 ± 10 g. All animals were regularly weighted in seven-day intervals (0, 7, 14, 21, 28 days). Rats were divided into five groups of six animals. They were fed on a monodiet containing wheat and different doses of selenium nanoparticles. Wheat naturally contained 0.03 mg Se/kg. Four groups of rats were fed with selenium nanoparticles in the dose 0.5, 1.5, 3.0 and 5.0 mg Se/kg per diet dose. The control group was fed with no selenium nanoparticles addition. Animals were placed in plastic boxes with metal grates measuring 40 × 60 × 20 cm. The experiment lasted for 28 days in which animals had access to feed and water ad libitum. During the experiment microclimatic condition (at 23 ± 1 °C), constant humidity (60%) and light regime (12 h light/12 h dark) were maintained. Maximum illumination was 200 lx. At the end of the experiment, all animals were put to death and samples of whole blood were collected, liver and duodenum were dissected out and used for biochemical and histological analysis.

### 2.4. Atomic Absorption Spectrometry (AAS)

Se concentration was determined as is described in our previous publication (Horky et al., 2016). Briefly, samples of whole blood were collected in a tube containing heparin as an anticoagulant. Then, 0.3 g of liver tissue and 0.5 g of blood were disintegrated in a muffle furnace (LAC, Rajhrad, Czech Republic) by dry method. Mineralised samples of liver and blood were analysed using a 280 Z Agilent Technologies atomic absorption spectrometer (Agilent, Santa Clara, CA, USA) with electrothermal atomisation. The measurement condition was 196 nm with Zeeman correction. 

### 2.5. Analysis of GPx, SOD, Glucose TOX and Activity of Liver Enzymes from Blood Samples

Blood parameters were determined according to commercial kits: GPx (Sigma Aldrich, St. Louis, USA); SOD (Sigma Aldrich, St. Louis, USA); TAS (RANDOX, Crumlin, Great Britain, cat. NX2332); Glucose(Biovendor-Laboratory medicine, Brno, Czechia, cat. 11601); ALP (Biovendor-Laboratory medicine, Brno, Czechia, cat. 10062); Total protein (Biuret) (Biovendor-Laboratory medicine, Brno, Czechia, cat. 12751); AST (Biovendor-Laboratory medicine, Brno, Czechia, cat. 10352); ALT (Biovendor-Laboratory medicine, Brno, Czechia, cat. 10452); Albumin (Biovendor-Laboratory medicine, Brno, Czechia, cat. 10001).

All measurements were performed spectrometrically using a Konelab T20xt biochemical analyser (Thermo Fisher Scientific, Waltham, MA, USA). The measurement conditions were set up according to the manufacturer’s assay protocol.

### 2.6. Histopathology Analysis 

In total, 10% neutral buffered formaldehyde was used for fixing tissues. Tissues were cut at 3.0 μm and placed onto Superfrost Plus slides (Leica, UK). All sections were oriented the same way. Tissue blocks were cut with remaining sections and dipped in wax (stored at 22 °C). Tissue sections were stained with haematoxylin and eosin following standard procedures. Photographs were taken using an inverted Olympus microscope IX 71 S8F-3 (Olympus, Tokyo, Japan). A 100x magnification was used for liver samples and 200x magnification for duodenum samples.

### 2.7. Data Analysis and Statistics 

The data were processed using STATISTICA.CZ, version 12.0 (TIBCO Software Palo Alto, USA). The results were expressed as mean ± standard deviation (SD). Normality was checked using the Shapiro-Wilk test. Statistical significance was determined using ANOVA and Scheffé’s test (one-way analysis).

## 3. Results

### 3.1. Dietary SeNP Effect on Growth Performance and Weight of Dissected Tissues

The growth performance of Wistar albino rats fed diets supplemented with dietary SeNPs (0, 1.5, 3, and 5 mg Se/kg) is presented in Table 1. 

The average weight and weight gain of rats showed no significant differences (*p* < 0.05) between tested groups. During dissection, the rat’s liver and duodenum were removed. Effects of different levels of dietary SeNPs on the weight of the liver and duodenum are presented in Table 2. No significant differences (*p* < 0.05) between tested groups have been observed.

### 3.2. Selenium Content in Blood and Liver Tissue

**Figure 2:**
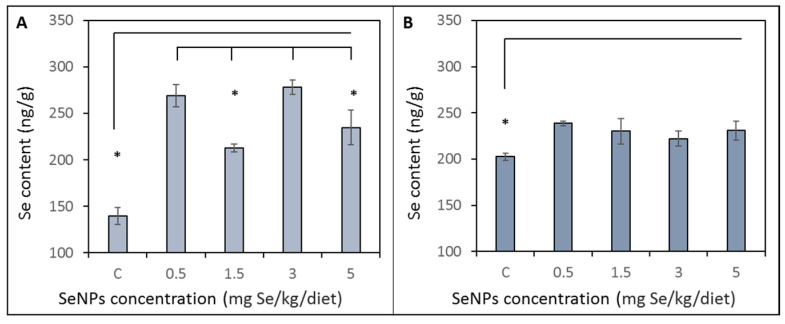
Selenium content obtained by AAS in (**A**) blood and (**B**) liver tissue. Rats were fed on a monodiet containing wheat and the different dose of selenium nanoparticles (0.5, 1.5, 3 and 5 mg Se/kg). Results are compared with the control group (C) of rats fed on a monodiet containing 0.03 mg Se/kg. * Mean values were significantly different (*p* < 0.05). Details of measurement are described in the Materials and Method section.

Se content in blood was significantly (*p* < 0.05) higher in all treated groups compared to the control group (139 ± 18 ng/g). Groups treated by 1.5 and 5 mg/Se/kg were significantly (*p* < 0.05) lower (212 ± 7 and 234 ± 30 ng/g, respectively) compared to other treated groups supplemented with 0.5 and 3 mg/Se/kg (268 ± 23 and 278 ± 18 ng/g, respectively). Results are shown in Figure 2A. With the increased dietary SeNP supplementation, liver Se concentrations significantly increased (*p* < 0.05) in the treated groups compared to the control group (200 ± 6 ng/g). The liver concentration of Se in the treated groups varied in the range from 222 to 238 ng/g. Results are shown in Figure 2B.

### 3.3. Biochemical Profile of Liver

**Figure 3:**
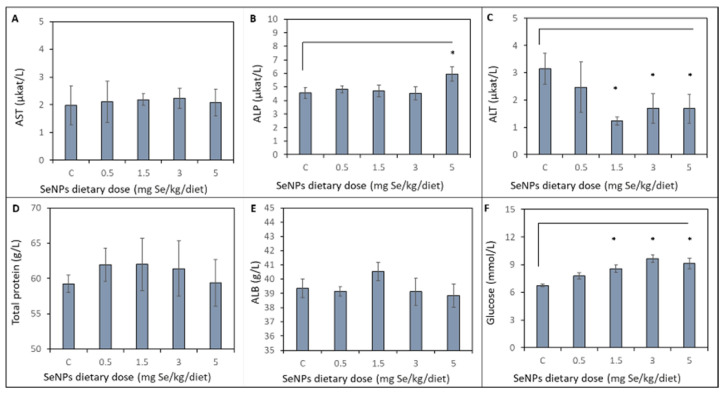
Biochemical profile of liver of control group (C) and after SeNP dietary intake determined from the blood. The activity of liver enzymes: (**A**) AST (**B**) ALP, (**C**) ALT. The concentration of (**D**) Total protein, (**E**) ALB and (**F**) Glucose. * Mean values were significantly different (***p***  <  0.05). Details of measurement are described in the Materials and Method section.

Effects of varying doses of dietary SeNP supplementation on biochemical profile of liver are shown in Figure 3. No significant (*p* < 0.05) differences were observed in activity of aspartate aminotransferase (AST) between the control and treated groups. The alkaline phosphatase (ALP) activity of the control group and treated groups (SeNP dietary dose 0.5, 1.5, 3 mg Se/kg) remains unchanged. A significant increase in ALP activity was observed in the SeNP-treated group with 5 mg Se/kg (*p* < 0.05) compared to the other treated groups. The ALP activity increased from average 4.65 ± 0.40 µkat/L to 5.95 ± 0.53 µkat/L in the group treated with the highest doses of SeNPs. A significant (p < 0.05) decrease in alanine aminotransferase (ALT) activity compared to the control group (3.15 ± 0.58 µkat/L) and 0.5 mg Se/kg SeNP-treated group (2.48 ± 0.92 µkat/L) was observed in the 1.5, 3 and 5 mg Se/kg SeNP-treated groups: 1.23 ± 0.14, 1.70 ± 0.54 and 1.68 ± 0.52 µkat/L, respectively. No significant (*p* < 0.05) differences were observed in total protein concentration between the control group and treated groups. Average total protein concentration was 60.80 ± 2.90 g/L. Albumin (ALB) concentration of the SeNP-treated groups was not significantly (*p* < 0.05) increased (40.53 ± 0.64 g/L) compared to the control group (39.36 ± 0.65 g/L) and other SeNP-treated groups. Glucose concentration significantly (*p* < 0.05) increased in 1.5, 3 and 5 mg Se/kg SeNP-treated groups (8.58 ± 0.42, 9.64 ± 0.38 and 9.13 ± 0.58 mmol/L, respectively) compared to the control group (6.75 ± 0.16 mmol/L) and 0.5 mg Se/kg SeNP-treated group (7.8 ± 0.33 mmol/L). 

### 3.4. Antioxidant Status of Rat´s Organism

**Figure 4:**
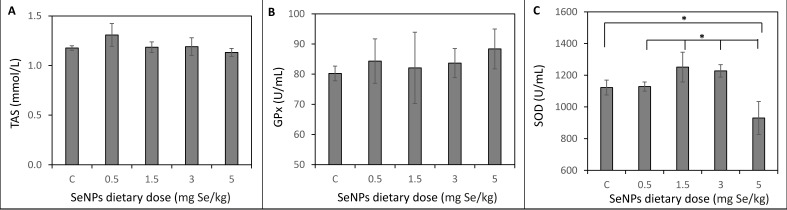
Antioxidant status of rats in the control group (C) and SeNP-enriched diet. (**A**) Total antioxidant status in blood, (**B**) GPx in blood and (**C**) SOD in the liver parenchyma. * Mean values were significantly different (*p*  <  0.05). Details of measurement are described in the Materials and Method section.

Results of the antioxidant status of the rat´s organism are shown in Figure 4. No significant (*p* < 0.05) differences were observed in total antioxidant status (TAS) between all tested groups. The average level of TAS was 1.20 ± 0.06 mmol/L. A slightly increasing trend of glutathione peroxidase (GPx) activity (from 80 to 88 U/mL) was observed with the rising levels of dietary SeNPs (from 0.5 to 5 mg Se/kg), but a significant (*p* < 0.05) effect was not observed. Superoxide dismutase (SOD) activity in liver was significantly decreased in the SeNP-treated group with 5 mg Se/kg (929 ± 103 U/mL) compared to the control group (1122 ± 47 U/mL) and the SeNP-treated groups 0.5, 1.5 and 3 mg Se/kg (1128 ± 28 U/mL, 1251 ± 94 U/mL, and 1227 ± 39 U/mL, respectively).

### 3.5. Histological Examination of the Liver and Duodenum 

**Figure 5:**
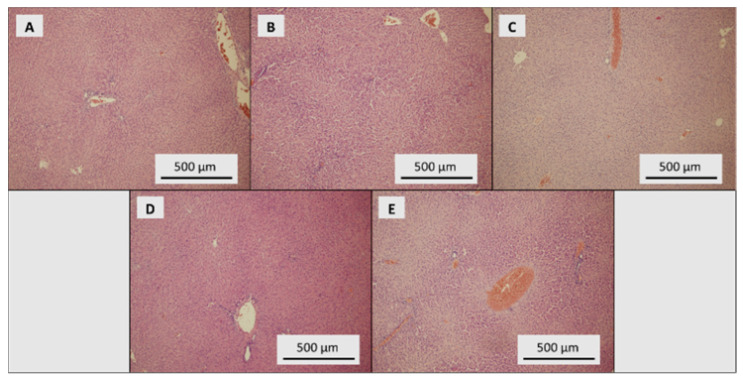
Histological examination of liver. (**A**) Control group, dose of SeNPs: (**B**) 0.5, (**C**) 1.5, (**D**) 3.0 and (**E**) 5 mg/kg/diet.

Liver parenchyma of the control group of rats showed signs of mild dystrophy with the preserved trabecular organisation and places with dilatation of portobilia. Intact structures without necrosis, inflammation and dysplasia were found (Figure 5A). Mildly dystrophic parenchyma with signs of congestion was observed in the SeNP-treated group with 0.5 mg Se/kg (Figure 5B). Almost the same features were found in the SeNP-treated group with 1.5 mg Se/kg, with occasional dilatation of portobilia (Figure 5C). The SeNP-treated group with 3.0 mg Se/kg showed mild dystrophic parenchyma, with the preserved trabecular organisation and portobiliary dilatations (Figure 5D). In the SeNP-treated group with 5.0 mg Se/kg, liver parenchyma with mild multifocal autolytic damage and congestion signs was found. No noticeable dystrophic changes were observed (Figure 5E).

In the control group, an intestinal profile with significant devastation of mucosal formations was observed, intestinal villi were low, damaged, deformed, and in some places were completely absent. Cup cells predominate in the lining, in the stroma of the villi, there were groups of mononuclear cellularization with isolated eosinophils. Tissue was without signs of inflammation, dysplasia or necrosis. Both sections of the intestine were identical (Figure 5A). The treated group with SeNPs 0.5 mg Se/kg showed deformed, irregular villi in both sections of the intestine with surface alteration, without crypt distortion or crypt abscesses and without a significantly increased number of intraepithelial leukocytes (Figure 5B). The profile of the intestine of the SeNP-treated group with 1.5 mg Se/kg showed shape variability of villi. The villi were swollen in places, with dense cellularization of the stroma. In some places, more numerous intraepithelial leukocytes occurred (up to 6 LEU/100 enterocytes). Crypts without signs of distortion and brindle cuticle without alteration were found (Figure 5C). In the SeNP-treated group with 3.0 mg Se/kg, the finding was identical to the previous group. In the aboral section, clusters of more frequent cellularization in the villi stroma, including eosinophils, were detected (Figure 5D). In both intestine sections, severe alteration of mucosal formations in the SeNP-treated group with 5.0 mg Se/kg were found. The aboral section was subtotally with the dominance of cup cells in the lining. The stroma of the villi showed frequent cellularization, including eosinophils (Figure 5E).

## 4. Discussion

Selenium is often discussed in connection with the role of the health status of organism. The suitable and sufficient form of Se is under discussion with the development of SeNPs. This promising solution must be carefully considered in the term of toxicity. In our previous study, we proved a positive effect of SeNPs on the antioxidant status of rats [16,17,18] and ejaculate quality of boars [19,20,21]. The conclusive effect of SeNPs has been proven by similar studies [22,23,24,25].

**Figure 6:**
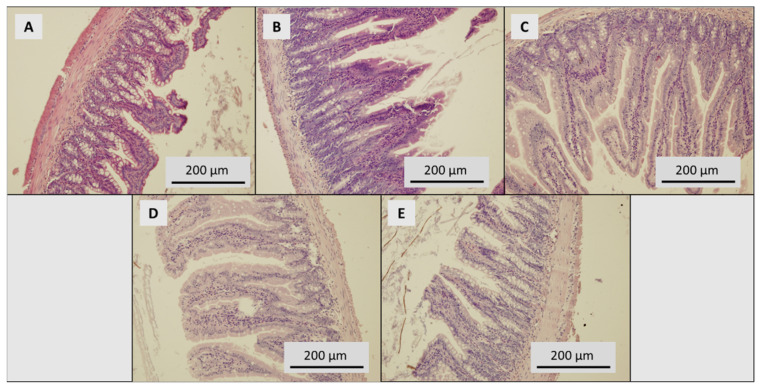
Histological examination of intestine. (**A**) Control group, dose of SeNPs: (**B**) 0.5, (**C**) 1.5, (**D**) 3.0 and (**E**) 5 mg/kg/diet.

In the presented study, we evaluated SeNP supplementation from non-toxic to toxic doses. The biochemical and antioxidant status of rat´s organism has been assessed as well as histopathology changes of liver parenchyma and intestinal tissue (Figure 6). The weight of experimental animals has been monitored during the experiment and after slaughter. Significant weight decrease has not been observed in treated groups compared to treated groups. Generally, in mammals, Se supplementation has been associated with increased weight gain due to improving nutrient availability [26]. The weight of dissected liver and duodenum related to body weight was also investigated in our study. Several authors described changes of liver and intestinal weight due to its damage as an organ involved in detoxification [27]. In our study, these effects have not been proved.

Selenium concentration of SeNP-treated groups 1.5 and 5 mg Se/kg showed a significant decrease in blood compared to the control and other treated groups. As blood is a transporting medium, the concentration of Se could be dramatically changed depending on food intake. Moreover, the buffering system of blood could be insufficient for higher doses of Se due to depletion of transport proteins, and non-deposited Se is excreted by urinary secretion [28,29]. On the other hand, the significantly higher concentration of Se in the SeNP-treated groups compared to the control group was observed in the liver, which isone of the the main depot organ for selenium [30].

Liver function tests have been employed to evaluate functional damage of the liver. Low doses of Sodium selenite in doses of 0.2 mg/kg/diet have shown to improve liver function after experimental liver injury [31]. It has been shown that higher therapeutic doses of Se caused a rapid increase in the level of liver enzymes leading to hepatic dysfunction [32]. Results in this study did not prove significant changes in liver function tests except for the increased level of ALP in the group treated by 5 mg Se/kg. In contrary, significantly decreased levels of ALT were observed in treated groups by 1.5, 3 and 5 mg Se/kg. Although decreased ALT activity is not directly associated with hepatocyte damage and increased enzyme release to the bloodstream, it may indicate dysfunction or abnormal liver function. Rapidly falling ALT levels have been associated with the exhaustion of the hepatocyte mass and terminal hepatic failure with a characteristic of coagulation [33,34]. A literature overview shows that high doses of Se lead to the destruction of hepatocytes; on the contrary, giving moderate doses reduces these enzymes’ activity under toxic load. Thus, therapeutic levels of Se may have a hepatoprotective effect. In our study, toxic effects on liver tissue at the biochemical level were not observed. Similar results were reported by Li et al. [35]. Compared to sodium selenite, biogenic SeNPs showed a protective effect on liver parenchyma and inhibiting effect for the elevation of the level of liver enzymes in carbon tetrachloride-induced liver damage in mice.

Evaluation of blood glucose levels showed a significant increase in experimental groups treated by 1.5, 3 and 5 mg Se/kg. Liver parenchyma metabolises dietary carbohydrates via phosphorylates glucose to glucose 6-phosphate inside the hepatocyte. The influence of Se supplementation on glucose metabolism is complex, and it is supposed to be reflected by non-linear U-shaped dose–response relationship [36]. Animal studies have indicated that Se supplementation may lead to hyperinsulinemia, insulin resistance, and glucose intolerance [37]. Kiersztan et al. reflected the different effect of inorganic (sodium selenite) and organic form (methylselenocysteine) on glucose homeostasis [38]. Different effects can be expected for various selenium nano forms. Non-toxic doses of SeNPs tend to decrease glucose levels in diabetic rats, as was described by several authors [39,40,41]. Al-Quraishy suggested a potential mechanism of action via the influence of malic enzyme, hexokinase and glucose-6-phosphate dehydrogenase activity [42]. Overall, SeNPs may affect glycaemic control at different levels of regulation dependent on Se form.

Selenium is one of the essential compounds that play a role in oxidative stress elimination. Thus, it could be expected that higher doses of Se affect TAC and levels of antioxidant enzymes, such as GPx and SOD. Surprisingly, the presented results have not shown significantly different levels of TAS and GPx compared to the control and treated groups [43]. In contrast to our results, Wang et al. [44] confirmed selenium activation of TAC, SOD and GPx in a dose-dependent manner of 0.1 and 0.2 mg Se/kg. It is noteworthy that most studies have utilised non-toxic levels of Se or SeNPs, and at these levels, SeNP supplementation can ameliorate the adverse effects of oxidative stress on the liver [45,46]. In the long-term period oxidative stress, or its sudden increase, has proved depletion of SOD activity [47,48]. In this study, the activity of SOD has been significantly decreased in the group of experimental animals treated by 5 mg Se/kg. On the other hand, lower doses of SeNPs (0.1 mg Se/kg) can cause a significant increase in SOD with an overall impact on improving the antioxidant system [49,50].

To eliminate the effect of natural micronutrients (other than Se), animals were fed on a monodiet. The weakness of this approach could lead to malnutrition demonstrated by mild damage of intestinal epithelium and liver parenchyma. Slight devastation of intestinal mucosa and villi, as well as mild dystrophy, has been observed in a histopathological examination of the control group. Significant dose-dependent deterioration was observed in groups treated with SeNPs. Our results are supported by Hey et al., who presented damage of selected tissues in supranutritional and nonlethal levels from 0.2 to 8 mg Se/kg [51]. Contrary to these results, no histological changes in the livers of rats exposed to SeNPs or selenite (0.05, 0.5 and 4mg Se/kg) have been observed in recently published results by Hadrup et al. [12].

SeNPs have displayed unique functionalities due to their nanoscale size. These properties are assigned to the lower surface area per unit volume, and, therefore, the nanoparticles are less interactive and release selenium more slowly. Moreover, the toxicity reported for elemental selenium (Se^0^) at nano size is lower than the toxicity of selenate (Se^II^), selenite (Se^IV^), or selenate (Se^VI^) ions. SeNPs seem to be less reactive than Se salts or organic compounds. 

## 5. Conclusions

Our study did not prove a significant toxic effect of SeNPs on the Wistar Albino rat animal model. The highest doses of SeNPs showed a decrease in weight gain (34 ± 11 g) compared to the control group (41 ± 9), a decrease in ALT activity (1.68 ± 0.52 µkat/L) compared to the control group (3.15 ± 0.58 µkat/L) and increase in blood glucose concentration (9.13 ± 0.58 mmol/L) compared to the control group (6.75 ± 0.16 mmol/L). No significant change was observed in the antioxidant status of the rats, only SOD activity in liver was significantly decreased in the SeNP-treated group with 5 mg Se/kg. Histopathological findings indicate damage to the liver parenchyma and intestinal epithelium in a dose-dependent manner. However, it can be concluded that short-term SeNP supplementation could be safe and beneficial in terms of Se deficiency or specific treatment. The challenge for further studies will be using SeNPs for targeted therapy or supplementation of selenium in its long-term deficiency. However, a pharmacokinetic investigation is required for these experiments.

## Figures and Tables

**Table 1:** Weight and weight gain (g) of Wistar albino rats during the feeding experiment (28 days).

Day	0	7	14	21	28	Weight Gain
Control group	155 ± 7	174 ± 11	195 ± 7	196 ± 8	197 ± 11	41 ± 9
SeNPs 0.5 mg/kg	151 ± 4	183 ± 4	191 ± 4	190 ± 4	198 ± 5	47 ± 4
SeNPs 1.5 mg/kg	149 ± 10	166 ± 10	176 ± 7	181 ± 8	183 ± 8	34 ± 9
SeNPs 3.0 mg/kg	160 ± 8	195 ± 7	200 ± 7	204 ± 7	204 ± 6	44 ± 7
SeNPs 5.0 mg/kg	154 ± 11	173 ± 11	179 ± 12	187 ± 11	188 ± 10	34 ± 11

**Table 2:** Weight (g) of liver and duodenum of Wistar albino rats at the end of the feeding experiment (28 days).

Sample	Liver	% of Live Weight	Duodenum	% of Live Weight
Control group	6.9 ± 0.9	3.7	7.3 ± 0.4	3.9
SeNPs 0.5 mg/kg	7.5 ± 0.4	3.8	7.0 ± 0.3	3.5
SeNPs 1.5 mg/kg	7.4 ± 0.6	4.0	6.6 ± 0.2	3.6
SeNPs 3.0 mg/kg	6.9 ± 0.4	3.3	7.0 ± 0.3	3.4
SeNPs 5.0 mg/kg	6.1 ± 0.6	3.2	7.4 ± 0.4	3.9

## Data Availability

Data available on request due to restrictions eg privacy or ethical.

## References

[1] Bodnar M., Konieczka P., Namiesnik J. (2012). The Properties, Functions, and Use of Selenium Compounds in Living Organisms. J. Environ. Sci. Health Part C Environ. Carcinogen. Ecotoxicol. Rev..

[2] Avery J.C., Hoffmann P.R. (2018). Selenium, Selenoproteins, and Immunity. Nutrients.

[3] Whanger P., Vendeland S., Park Y.C., Xia Y.M. (1996). Metabolism of subtoxic levels of selenium in animals and humans. Ann. Clin. Lab. Sci..

[4] Seko Y., Imura N. (1997). Active oxygen generation as a possible mechanism of selenium toxicity. Biomed. Environ. Sci..

[5] Bhattacharjee A., Basu A., Bhattacharya S. (2019). Selenium nanoparticles are less toxic than inorganic and organic selenium to mice in vivo. Nucl. Ind..

[6] Chaudhary S., Umar A., Mehta S.K. (2016). Selenium nanomaterials: An overview of recent developments in synthesis, properties and potential applications. Prog. Mater. Sci..

[7] Khurana A., Tekula S., Saifi M.A., Venkatesh P., Godugu C. (2019). Therapeutic applications of selenium nanoparticles. Biomed. Pharm..

[8] Skalickova S., Milosavljevic V., Cihalova K., Horky P., Richtera L., Adam V. (2017). Selenium nanoparticles as a nutritional supplement. Nutrition.

[9] Wadhwani S.A., Shedbalkar U.U., Singh R., Chopade B.A. (2016). Biogenic selenium nanoparticles: Current status and future prospects. Appl. Microbiol. Biotechnol..

[10] Indumathy M., Raj S.S., Arumugham I.M., Kumar R.P. (2020). Assessment of Toxicity of Selenium Nanoparticle Varnish Using HepG2 Cell Lines: In vitro Study. J. Pharm. Res. Int..

[11] Qamar N., John P., Hatti A.B. (2020). Toxicological and Anti-Rheumatic Potential of Trachyspermum ammi Derived Biogenic Selenium Nanoparticles in Arthritic Balb/c Mice. Int. J. Nanomed..

[12] Hadrup N., Loeschner K., Mandrup K., Ravn-Haren G., Frandsen H.L., Larsen E.H., Lam H.R., Mortensen A. (2019). Subacute oral toxicity investigation of selenium nanoparticles and selenite in rats. Drug Chem. Toxicol..

[13] Kumar N., Krishnani K.K., Singh N.P. (2018). Comparative study of selenium and selenium nanoparticles with reference to acute toxicity, biochemical attributes, and histopathological response in fish. Environ. Sci. Pollut. Res..

[14] Gangadoo S., Dinev I., Willson N.L., Moore R.J., Chapman J., Stanley D. (2020). Nanoparticles of selenium as high bioavailable and non-toxic supplement alternatives for broiler chickens. Environ. Sci. Pollut. Res..

[15] Caracciolo G., Farokhzad O.C., Mahmoudi M. (2017). Biological Identity of Nanoparticles In Vivo: Clinical Implications of the Protein Corona. Trends Biotechnol..

[16] Urbankova L., Pribilova M., Horky P. The Influence of Different Forms of Selenium on Vitality of Laboratory Rats. Proceedings of the 26th International PhD Students Conference for Undergraduate and Postgraduate (MendelNet).

[17] Urbankova L., Horky P., Skladanka J., Pribilova M., Smolikova V., Nevrkla P., Cernei N., Lackova Z., Hedbavny J., Ridoskova A. (2018). Antioxidant status of rats’ blood and liver affected by sodium selenite and selenium nanoparticles. PeerJ.

[18] Horky P., Ruttkay-Nedecky B., Nejdl L., Richtera L., Cernei N., Pohanka M., Kopel P., Skladanka J., Hloucalova P., Slama P. (2016). Electrochemical Methods for Study of Influence of Selenium Nanoparticles on Antioxidant Status of Rats. Int. J. Electr. Sci..

[19] Horky P., Jancikova P., Sochor J., Hynek D., Chavis G.J., Ruttkay-Nedecky B., Cernei N., Zitka O., Zeman L., Adam V. (2012). Effect of Organic and Inorganic Form of Selenium on Antioxidant Status of Breeding Boars Ejaculate Revealed by Electrochemistry. Int. J. Electrochem. Sci..

[20] Horky P., Skladanka J., Nevrkla P., Slama P. (2016). Effect of diet supplemented with antioxidants (selenium, copper, vitamins e and c) on antioxidant status and ejaculate quality of breeding boars. Ann. Anim. Sci..

[21] Horky P., Sochor J., Skladanka J., Klusonova I., Nevrkla P. (2016). Effect of selenium, vitamins E and C on antioxidant potential and quality of boar ejaculate. J. Anim. Feed Sci..

[22] Pardechi A., Tabeidian S.A., Habibian M. (2020). Comparative assessment of sodium selenite, selenised yeast and nanosized elemental selenium on performance response, immunity and antioxidative function of broiler chickens. It. J. Anim. Sci..

[23] Shen X.Y., Huo B., Gan S.Q. (2020). Effects of Nano-Selenium on Antioxidant Capacity in Se-Deprived Tibetan Gazelle (Procapra picticaudata) in the Qinghai-Tibet Plateau. Biol. Trace Element Res..

[24] Lee J., Hosseindoust A., Kim M., Kim K., Choi Y., Lee S., Cho H., Chae B. (2020). Supplemental hot melt extruded nano-selenium increases expression profiles of antioxidant enzymes in the livers and spleens of weanling pigs. Anim. Feed Sci. Technol..

[25] Zheng Y.L., Dai W.Z., Hu X.L., Hong Z.P. (2020). Effects of dietary glycine selenium nanoparticles on loin quality, tissue selenium retention, and serum antioxidation in finishing pigs. Anim. Feed Sci. Technol..

[26] Reed J.J., Ward M.A., Vonnahme K.A., Neville T.L., Julius S.L., Borowicz P.P., Taylor J.B., Redmer D.A., Grazul-Bilska A.T., Reynolds L.P. (2007). Effects of selenium supply and dietary restriction on maternal and fetal body weight, visceral organ mass and cellularity estimates, and jejunal vascularity in pregnant ewe lambs. J. Anim. Sci..

[27] Strubelt O., Kremer J., Tilse A., Keogh J., Pentz R., Younes M. (1996). Comparative studies on the toxicity of mercury, cadmium, and copper toward the isolated perfused rat liver. J. Toxicol. Environ. Health.

[28] Hall J.A., Bobe G., Nixon B.K., Vorachek W.R., Hugejiletu, Nichols T., Mosher W.D., Pirelli G.J. (2014). Effect of transport on blood selenium and glutathione status in feeder lambs. J. Anim. Sci..

[29] Zheng S.F., Xing H.J., Zhang Q.J., Xue H., Zhu F.T., Xu S.W. (2019). Pharmacokinetics of Sodium Selenite Administered Orally in Blood and Tissues of Selenium-Deficient Ducklings. Biol. Trace Element Res..

[30] Shang N.N., Wang X.F., Shu Q.M., Wang H., Zhao L.N. (2019). The Functions of Selenium and Selenoproteins Relating to the Liver Diseases. J. Nanosci. Nanotechnol..

[31] Ozardali I., Bitiren M., Karakilcik A.Z., Zerin M., Aksoy N., Musa D. (2004). Effects of selenium on histopathological and enzymatic changes in experimental liver injury of rats. Exp. Toxicol. Pathol..

[32] Zwolak I., Zaporowska H. (2012). Selenium interactions and toxicity: A review Selenium interactions and toxicity. Cell Biol. Toxicol..

[33] Nardo B., Puviani L., Caraceni P., Pacile V., Bertelli R., Beltempo P., Cavallari G., Chieco P., Pariali M., Pertosa A.M. (2006). Portal vein arterialization for the treatment of post resection acute liver failure in the rat. Transpl. Proc..

[34] Schemitt E.G., Hartmann R.M., Colares J.R., Licks F., Salvi J.O., Marroni C.A., Marroni N.P. (2019). Protective action of glutamine in rats with severe acute liver failure. World J. Hepatol..

[35] Li B.Z., Li D., Jing W.X., Fan J.H., Dahms H.U., Lee S.C., Wang L. (2017). Biogenic selenium and its hepatoprotective activity. Sci. Rep..

[36] Wang X.L., Yang T.B., Wei J., Lei G.H., Zeng C. (2016). Association between serum selenium level and type 2 diabetes mellitus: A non-linear dose-response meta-analysis of observational studies. Nutr. J..

[37] Zeng M.S., Li X., Liu Y., Zhao H., Zhou J.C., Li K., Huang J.Q., Sun L.H., Tang J.Y., Xia X.J. (2012). A high-selenium diet induces insulin resistance in gestating rats and their offspring. Free Rad. Biol. Med..

[38] Kiersztan A., Lukasinska I., Baranska A., Lebiedzinska M., Nagalski A., Derlacz R.A., Bryla J. (2007). Differential effects of selenium compounds on glucose synthesis in rabbit kidney-cortex tubules and hepatocytes. In vitro and in vivo studies. J. Inorganic Biochem..

[39] Ebokaiwe A.P., Okori S., Nwankwo J.O., Ejike C., Osawe S.O. (2020). Selenium nanoparticles and metformin ameliorate streptozotocin-instigated brain oxidative-inflammatory stress and neurobehavioral alterations in rats. Naunyn Schmiedebergs Arch. Pharmacol..

[40] Deng W.J., Wang H., Wu B.J., Zhang X.W. (2019). Selenium-layered nanoparticles serving for oral delivery of phytomedicines with hypoglycemic activity to synergistically potentiate the antidiabetic effect. Acta Pharm. Sinica B.

[41] Liu Y.T., Zeng S.G., Liu Y.X., Wu W.J., Shen Y.B., Zhang L., Li C., Chen H., Liu A.P., Shen L. (2018). Synthesis and antidiabetic activity of selenium nanoparticles in the presence of polysaccharides from Catathelasma ventricosum. Int. J. Biol. Macromol..

[42] Al-Quraishy S., Dkhil M.A., Moneim A.E.A. (2015). Anti-hyperglycemic activity of selenium nanoparticles in streptozotocin-induced diabetic rats. Int. J. Nanomed..

[43] Eid S.Y., El-Zaher H.M., Emara S.S., Farid O.A., Michael M.I. (2019). Nano selenium treatment effects on thyroid hormones, immunity and antioxidant status in rabbits. World Rabbit Sci..

[44] Wang Z.N., Li H., Tang H., Zhang S.J., Pauline M., Bi C.L. (2020). Short Communication: Effects of Dietary Selenium Supplementation on Selenium Deposition and Antioxidant Status in Postpartum Mice. Biol. Trace Element Res..

[45] Zidkova J., Melcova M., Mlejnek P., Zidek V., Szakova J., Koplik R., Mestek O. (2015). The effect of dietary selenium on antioxidative status in rats. Ann. Nutr. Metab..

[46] Nasirpour M., Sadeghi A.A., Chamani M. (2017). Effects of nano-selenium on the liver antioxidant enzyme activity and immunoglobolins in male rats exposed to oxidative stress. J. Livestock Sci..

[47] Culotta V.C. (2000). Superoxide dismutase, oxidative stress, and cell metabolism. Current Topics in Cellular Regulation.

[48] Lucca G., Comim C.M., Valvassori S.S., Reus G.Z., Vuolo F., Petronilho F., Dal-Pizzol F., Gavioli E.C., Quevedo J. (2009). Effects of chronic mild stress on the oxidative parameters in the rat brain. Neurochem. Int..

[49] Guo L.L., Xiao J.Y., Liu H.J., Liu H.M. (2020). Selenium nanoparticles alleviate hyperlipidemia and vascular injury in ApoE-deficient mice by regulating cholesterol metabolism and reducing oxidative stress. Metallomics.

[50] Hamza R.Z., Diab A.E.-A.A. (2020). Testicular protective and antioxidant effects of selenium nanoparticles on Monosodium glutamate-induced testicular structure alterations in male mice. Toxicol. Rep..

[51] He Y.D., Chen S.Y., Liu Z.X., Cheng C., Li H., Wang M.Q. (2014). Toxicity of selenium nanoparticles in male Sprague-Dawley rats at supranutritional and nonlethal levels. Life Sci..

